# Commentary on “the middle fossa approach with self-drilling screws: a novel technique for BONEBRIDGE implantation”

**DOI:** 10.1186/s40463-019-0377-0

**Published:** 2019-11-05

**Authors:** C. Carnevale, M. Tomás-Barberán, G. Til-Pérez, E. Enchev, P. Sarría-Echegaray

**Affiliations:** Otolaryngology Head and Neck Surgery Department, Son Espases University Hospital, Carretera Valldemosa, 79. 07210 Servicio ORL, Palma de Mallorca, Islas Balears Spain

To the Editor:

We read with great interest the article titled “The middle fossa approach with self-drilling screws: a novel technique for BONEBRIDGE implantation” by You et al. [1]. In this study with 40 patients, the authors concluded that middle fossa approach with self drilling screws is a safe alternative to Bonebridge implantation, with no complications after an average follow up of 29 months. We consider that this study is properly conducted and highlights many of the benefits of middle fossa approach. The detailed description of the surgical technique and the large serie of patients make this article an excellent reference for otologic surgeons opting to use this approach.

In spite of this, the middle fossa approach technique was described in 14 patients with similar results after a follow up of 6–45 months in a previously study published by our group [2]. It was observed that the use of a neurodrill to create the bone bed makes this approach safer and easier, shortening the operative time and reducing the risk of damage to the dura mater and sigmoid sinus when compared with a classic otologic drill. With respect to the audiologic outcomes, as You et al. mention, we didn’t observe any difference between the three possible approaches used for Bonebridge implantation, reporting an average functional gain of 33.46dB [3], comparable to what has been reported in the literature [4, 5].

We agree with the conclusions of You et al. [1], and actually we consider this technique as a novel safe approach especially in patients where a classic presigmoid approach is not feasible. Furthermore it is technically easy and reproducible, with lower rate of complications and short surgical time. For all these reasons we believe that this approach could be the first choice technique for Bonebridge implantation, but further studies with larger series are needed to confirm our hypothesis.

## Data Availability

Not applicable.
